# Influence of dual antiplatelet therapy duration on neointimal condition after second-generation drug-eluting stent implantation

**DOI:** 10.1007/s12928-021-00765-8

**Published:** 2021-02-26

**Authors:** Yutaka Goryo, Teruyoshi Kume, Hiroshi Okamoto, Ai Kawamura, Kenzo Fukuhara, Tomoko Tamada, Terumasa Koyama, Koichiro Imai, Ryotaro Yamada, Yoji Neishi, Shiro Uemura

**Affiliations:** Department of Cardiology, Kawasaki Medical School, 577 Matsushima, Kurashiki, Okayama 701-0192 Japan

**Keywords:** Percutaneous coronary intervention, Drug-eluting stent, Antiplatelet therapy, Optical coherence tomography

## Abstract

Guidelines recommend shorter duration (1–12 months) for dual antiplatelet therapy (DAPT) in the second-generation drug-eluting stent (DES) era. However, whether shorter DAPT duration affects stent strut conditions and neointimal characteristics at mid-term follow-up remains uncertain. Therefore, we studied the relation between DAPT duration and vascular healing response as assessed by optical coherence tomography (OCT). This study was retrospective observational study. Participants comprised 64 patients who underwent serial OCT at both 9 and 18 months after DES implantation. All patients received DAPT until the 9-month follow-up then were divided into two groups: 49 patients who continued DAPT (longer DAPT group); and 15 patients who stopped taking the P2Y12 inhibitor and were treated with aspirin alone (shorter DAPT group) at the 18-month follow-up. Using OCT, we evaluated and compared stent strut conditions and neointimal characteristics between groups at both 9 and 18 months after stent implantation. Baseline clinical and procedural parameters were mostly similar between groups. At the 18-month follow-up, no in-stent thrombus assessed by OCT was observed in either group. No significant differences in OCT characteristics or measurements of neointima were seen between groups at 9- or 18-month follow-ups. Neointimal volume increased from 9 to 18 months in both groups, with a similar degree of neointimal proliferation in both groups (shorter DAPT group, 0.23 ± 0.29 mm^3^/mm; longer DAPT group, 0.19 ± 0.27 mm^3^/mm; *P* = 0.56). In conclusion, interrupting DAPT 9 months after second-generation DES implantation did not affect the development of in-stent thrombus, neointimal proliferation or stent strut coverage at 18-month follow-up compared with continuing DAPT.

## Introduction

Stent implantation has developed as a treatment for coronary artery disease. Bare metal stents (BMS) successfully restore the acute vessel lumen, but long-term outcomes are often compromised by in-stent restenosis (ISR). Drug-eluting stents (DES) markedly reduce ISR compared with BMS and indications for percutaneous coronary intervention (PCI) have widened. In the second-generation DES era, some clinical trials suggested the superiority of DES over BMS even with in-stent thrombosis and patients with the acute coronary syndrome (ACS) [1–3].

A previous study showed that longer administration of dual antiplatelet therapy (DAPT) prevented thrombotic adverse events, but increased the risk of hemorrhagic complications [4]. On the other hand, some recent papers have shown that shorter DAPT duration did not increase thrombotic events, particularly with the use of second-generation DES [1, 5, 6]. The guideline thus recommends a shorter duration of DAPT after DES implantation, particularly for patients at high risk of bleeding [7, 8]. However, differences in stent strut condition between patients with shorter and longer DAPT administration at mid-term after DES implantation remain unclear. The purpose of this study was to evaluate the influence of DAPT duration on neointimal condition after second-generation DES implantation using optical coherence tomography (OCT).

## Methods

### Study population

**Fig. 1:**
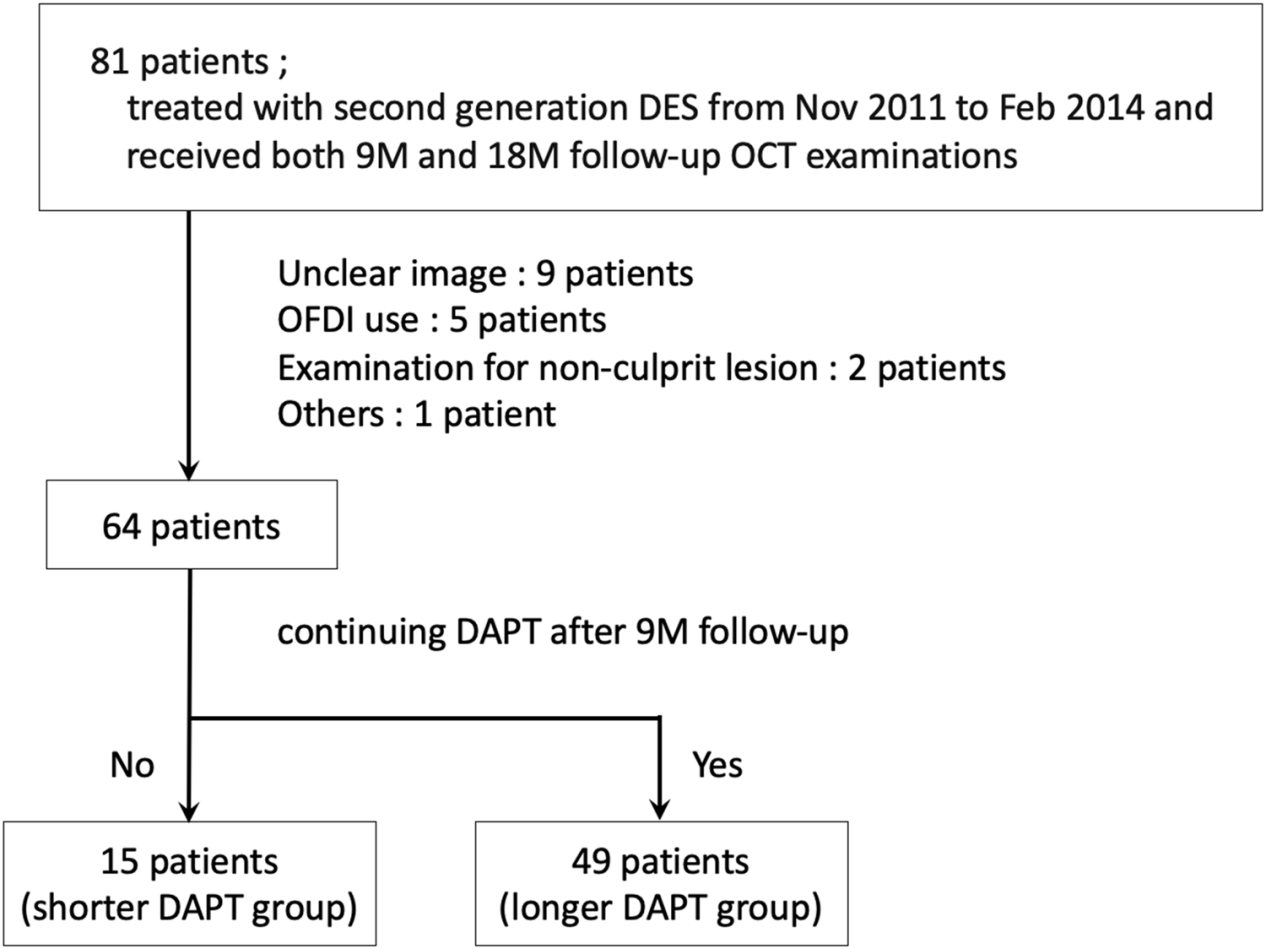
Patients flow chart. *DES* drug-eluting stent, *M* months, *OCT* optical coherence tomography, *OFDI* optical frequency domain imaging, *DAPT* dual antiplatelet therapy

Participants comprised 64 consecutive patients who underwent PCI with second-generation DES as a treatment for de novo lesions and were subsequently followed-up with OCT examinations at Kawasaki Medical School Hospital from November 2011 to February 2014 (Fig. 1). The interventional strategy and stent selection were left to the discretion of the operator. Follow-up OCT (ILUMIEN or ILUMIEN OPTIS OCT Intravascular Imaging System; St. Jude Medical, St. Paul, MN) were performed at 9 months and 18 months after DES implantation. Second-generation DES included everolimus-eluting stents (CoCr-EES, Xience™; Abbott Vascular, Santa Clara, CA or PtCr-EES, PROMUS™; Boston Scientific, Natick, MA), biolimus-eluting stents (BES, Nobori^®^; Terumo Corporation, Tokyo, Japan), and zotarolimus-eluting stents (ZES: Resolute™, Medtronic, Santa Rosa, CA).

DAPT with 100 mg of aspirin and either 75 mg of clopidogrel or 200 mg of ticlopidine was started at PCI and continued at least until the 9-month follow-up angiography. We classified patients into two groups: the shorter DAPT group with interruption of DAPT after the 9-month follow-up; and the longer DAPT group with the continuation of DAPT until the 18-month follow-up. The duration of DAPT was left to the doctor’s discretion. In addition, we calculated the PRECISE-DAPT score at index PCI and the DAPT score at 9-month follow-up [9, 10]. Patients with unclear OCT images due to insufficient blood removal or artifacts at follow-up coronary angiographies were excluded from analysis. In-stent restenosis (ISR) was defined angiographical more than 50% stenosis of the stented segment at follow-up periods.

This study was performed in compliance with the Declaration of Helsinki with regard to investigations involving human participants. The study protocol was approved by the ethics committee at Kawasaki Medical School, and written informed consent was obtained from each patient prior to enrolment.

### OCT imaging and analysis

**Fig. 2:**
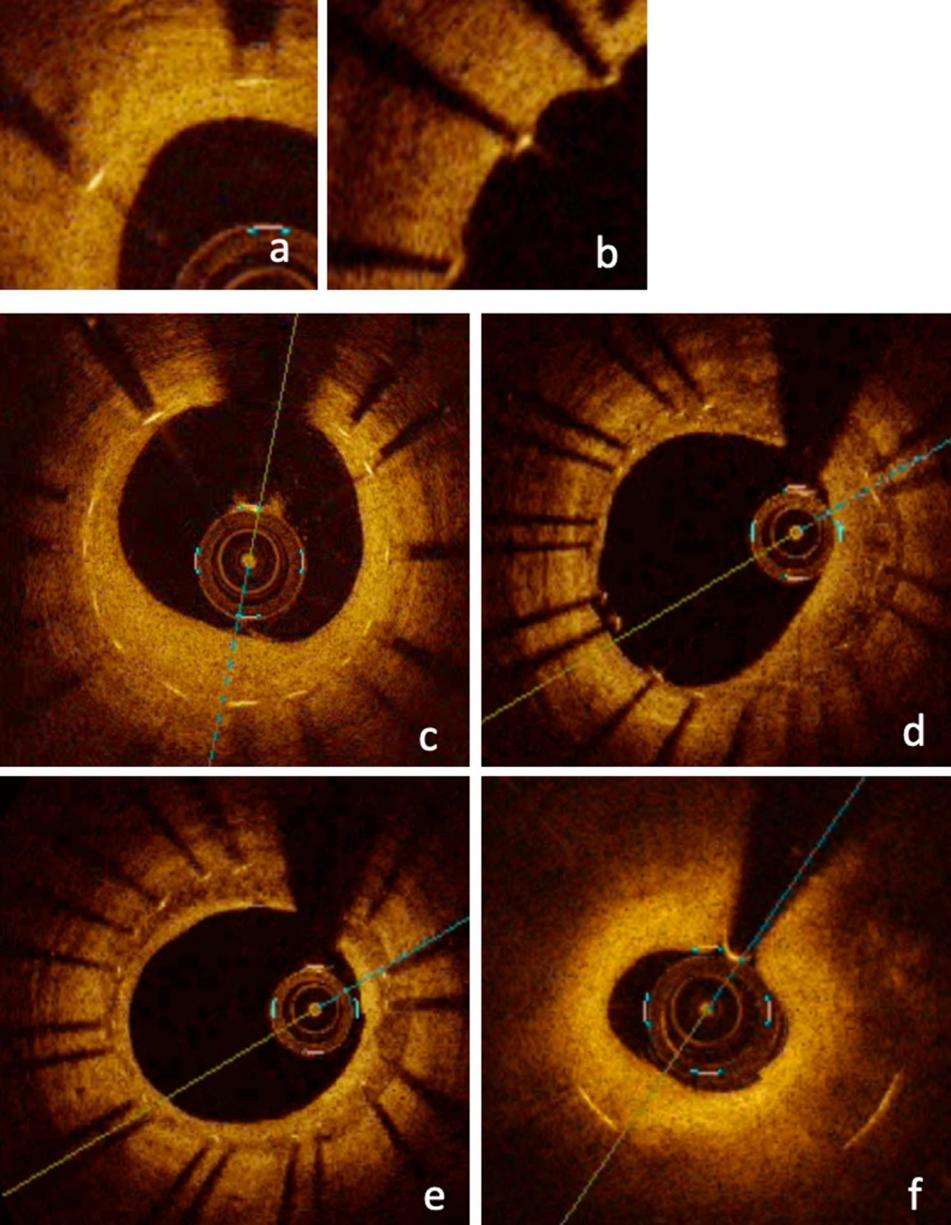
Representative images of optical coherence tomography. **a** Covered struts. **b** Uncovered struts. **c** Homogeneous pattern. **d** Heterogeneous pattern. **e** Layered pattern. **f** Neoatherosclerosis (with lipid-laden neointimal)

At follow-up angiographies, OCT was performed after intracoronary administration of nitroglycerin (0.2–0.3 mg). The OCT catheter was placed > 10 mm distal to the stented lesion and pulled back using an automatic pullback system. Cross-sectional OCT images of stented segments at 1-mm intervals were analyzed. We evaluated the neointimal coverage of each stent strut, neointimal volume, neointimal tissue characteristics and presence of thrombus. A covered strut was defined as a neointimal thickness (NIT), representing the distance between the center of the strut and the neointimal surface, > 30 μm (Fig. 2) [11, 12]. Neointimal volume was calculated by adding each neointimal area (stent area minus lumen area) throughout the stented segment. Neointimal volume index was determined as the neointimal volume divided by the length of the stented lesion. We evaluated the neointimal characteristics at the minimum lumen area (MLA) site of the stented segment and classified into four patterns: homogeneous, heterogeneous, layered and neoatherosclerosis (NA) [13]. NA was defined as the presence of lipid, calcification, thin-cap fibroatheroma, neovascularization and intimal rupture in the neointima, as mentioned in previous studies [14, 15]. Thrombus was defined as a mass protruding into the lumen with an irregular surface [16]. These OCT findings were compared between shorter DAPT and longer DAPT groups.

### Statistical analysis

Continuous variables are presented as mean ± standard deviation or median and interquartile range and were compared using a two-tailed unpaired *t* test or Wilcoxon rank-sum test. Categorical variables are expressed as percentages and were compared using the Chi-square test. Values of *P* < 0.05 were considered statistically significant. All statistical analyses were conducted using JMP for Mac version 11.0 (SAS Institute, Cary, NC).

## Results

A total of 64 patients (64 lesions) were studied, with 15 patients in the shorter DAPT group and 49 patients in the longer DAPT group. This study was retrospective observational study and guidelines of the Japanese circulation society recommended that DAPT duration after DES implantation was more than 12 months when analyzed cases underwent PCI [17]. Therefore, we continued DAPT for most of the cases without revascularization at 9-month follow-up angiography. However, recent studies reported the safety and effectiveness of short DAPT duration after DES implantation [1, 5, 6] and the duration of DAPT was left to the doctor’s discretion in this study.

Baseline clinical characteristics are shown in Table 1. In terms of risk factors, no significant differences were evident between groups, but a tendency was seen toward hypertension being more frequent in the longer DAPT group than in the shorter DAPT group. DAPT score and PRICISE-DAPT score did not differ significantly between groups. Pharmacotherapies excluding antiplatelet drugs at the 18-month follow-up are shown in Table 2. Angiotensin II receptor blockers, β-blockers and calcium channel blockers were used more frequently in the longer DAPT group than in the shorter DAPT group because of the patients’ comorbidity of hypertension. All patients in the shorter DAPT group stopped taking a P2Y12 inhibitor (not aspirin) when changing from DAPT to SAPT. In longer DAPT group, there were three patients (6%) who took ticlopidine and the remaining patients took clopidogrel as a P2Y12 inhibitor.

**Table 1:** Patients characteristics

	Shorter DAPT group	Longer DAPT group	*P* value
No. of patients	15	49	
Male gender, *n* (%)	11 (73.3)	39 (80.0)	0.61
Age, years old	67.8 ± 9.44	67.3 ± 10.5	0.92
Follow up, days			
0–9 M	275 ± 19.5	290 ± 29.6	0.04
0–18 M	550 ± 20.9	590 ± 53.6	< 0.01
Risk factors			
Hypertension, *n* (%)	9 (60)	41 (83.7)	0.05
Dyslipidemia, *n* (%)	11 (73.3)	39 (79.6)	0.61
Diabetes Mellitus, *n* (%)	4 (26.7)	19 (38.8)	0.39
Smoker, *n* (%)			0.74
Current	2 (13.3)	11 (22.5)	
Past	4 (26.7)	11 (22.5)	
BMI, kg/m^2^	24.0 ± 3.54	24.8 ± 3.76	0.45
Family history, *n* (%)	3 (20)	7 (14.3)	0.59
ACS, *n* (%)	9 (60)	15 (30.6)	0.12
Previous cardiac surgery, *n* (%)	0 (0)	2 (4.1)	0.43
Hemodialysis, *n* (%)	0 (0)	2 (4.1)	0.43
PRECISE-DAPT score at PCI	17.4 ± 12.9	18.2 ± 10.5	0.64
DAPT score at 9 M f/u	1.46 ± 1.24	1.26 ± 1.41	0.68

Values are expressed as mean standard deviation (SD) or *n* (%)

*BMI* body mass index, *ACS* acute coronary syndrome, *PCI* percutaneous coronary intervention, *DAPT* dual antiplatelet therapy, *M* months, *f/u* follow-up

**Table 2:** Medications at 18 M follow-up

	Shorter DAPT group	Longer DAPT group	*P* value
EPA, *n* (%)	1 (6.7)	9 (18.4)	0.27
Statin, *n* (%)	11 (73.3)	37 (75.5)	0.86
Vasodilator, *n* (%)	11 (73.3)	28 (57.1)	0.26
CCB, *n* (%)	1 (6.7)	20 (40.8)	0.01
β-blocker, *n* (%)	2 (13.3)	20 (40.8)	0.049
ACE-I, *n* (%)	5 (33.3)	10 (20.4)	0.30
ARB, *n* (%)	2 (13.3)	26 (53.1)	< 0.01
Diuretic, *n* (%)	3 (20)	7 (14.3)	0.59
Anticoagulation, *n* (%)	3 (20)	4 (8.2)	0.20

Values are numbers (%) of patients

*M* months, *EPA* eicosapentaenoic acid, *CCB* calcium channel blocker, *ACE-I* angiotensin-converting enzyme inhibitor, *ARB* angiotensin II receptor blocker

Lesion and procedural characteristics are listed in Table 3. Target vessel, selected stent type and stent size were similar in both groups. Frequency of ISR did not differ significantly between groups at either follow-up.

**Table 3:** Lesion and procedural characteristics

	Shorter DAPT group	Longer DAPT group	*P* value
No. of target lesion, *n* (%)			0.89
LAD	10 (66.7)	30 (61.2)	
LCX	1 (6.7)	5 (10.2)	
RCA	4 (26.7)	14 (28.6)	
Stent type, *n* (%)			0.86
CoCr-EES	6 (40)	17 (30.4)	
PtCr-EES	5 (33.3)	18 (32.1)	
BES	3 (20)	15 (26.8)	
ZES	1 (6.7)	6 (10.7)	
No. of stent per lesion	1.07 ± 0.26	1.12 ± 0.33	0.56
Stent diameter, mm	2.75 ± 0.25	2.82 ± 0.33	0.56
Stent length, mm	24.9 ± 5.37	22.6 ± 6.96	0.18
Imaging device at index PCI, *n* (%)			0.28
OCT/OFDI	12 (80)	32 (65.3)	
IVUS	3 (20)	17 (34.7)	
ISR at 9 M f/u, *n* (%)	0 (0)	1 (2)	0.58
ISR at 18 M f/u, *n* (%)	1 (6.7)	2 (4.1)	0.68

Values are expressed as mean standard deviation (SD) or *n* (%)

*LAD* left anterior descending artery, *LCX* left circumflex artery, *RCA* right coronary artery, *CoCr* cobalt chrome, *PtCr* platinum chromium, *EES* everolimus-eluting stent, *BES* biolimus-eluting stent, *ZES* zotarolimus-eluting stent, *OCT* optical coherence tomography, *OFDI* optical frequency domain imaging, *IVUS* intravascular ultrasound, *ISR* in-stent restenosis, *M* months, *f/u* follow-up

### OCT findings at follow-up

OCT findings at 9- and 18-month follow-ups are shown in Tables 4 and 5. As for neointimal tissue characteristics at MLA site, no significant differences were evident between groups during these periods, with each group predominantly showing a homogeneous pattern. Most stent struts were covered with neointima in both groups. Evidence of thrombus was detected in one case from the longer DAPT group at 9-month follow-up but had disappeared by the 18-month follow-up.

**Table 4:** OCT findings at 9 M follow-up angiography

	Shorter DAPT group	Longer DAPT group	*P* value
Neointimal characteristics at MLA site			0.71
Neoatherosclerosis	0 (0)	2 (4.1)	
Homogeneous	14 (93.3)	43 (87.8)	
Heterogeneous	1 (6.7)	4 (8.1)	
Layered	0 (0)	0 (0)	
Thrombus, *n* (%)	0 (0)	1 (2)	0.58
Average of stent area, mm^3^/mm	7.38 ± 2.69	6.99 ± 2.01	0.92

Values are expressed as mean standard deviation (SD) or *n* (%)

*OCT* optical coherence tomography, *M* months, *MLA* minimum lumen area

**Table 5:** OCT findings at 18 M follow-up angiography

	Shorter DAPT group	Longer DAPT group	*P* value
Neointimal characteristics at MLA site			0.54
Neoatherosclerosis	2 (13.3)	2 (4.1)	
Homogeneous	12 (80)	44 (89.8)	
Heterogeneous	1 (6.7)	2 (4.1)	
Layered	0 (0)	1 (2)	
Thrombus, *n* (%)	0 (0)	0 (0)	1.00
Average of stent area, mm^3^/mm	7.04 ± 2.26	6.82 ± 1.96	0.92

Values are expressed as mean standard deviation (SD) or *n* (%)

*OCT* optical coherence tomography, *M* months, *MLA* minimum lumen area

**Fig. 3:**
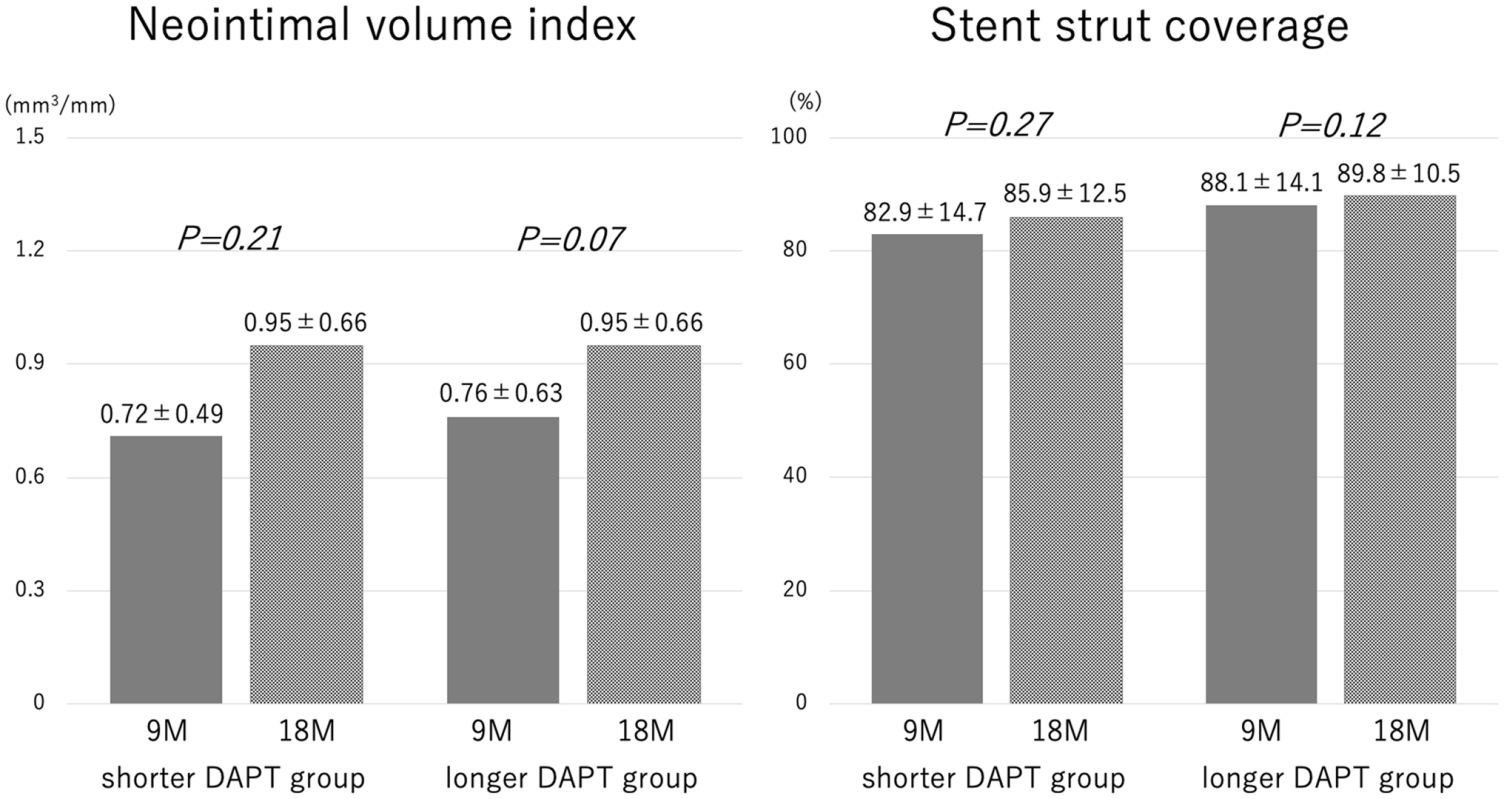
Neointimal volume index and stent strut coverage at 9-month and 18-month follow-up in shorter DAPT and longer DAPT group. *M* months. Values are expressed as mean ± standard deviation (SD) or *n* (%)

Changes in OCT findings between 9- and 18-month follow-ups are shown in Table 6 and Fig. 3. In both groups, neointimal volume and stent strut coverage tended to be increased (no significant difference) and increments in neointimal proliferation were similar.

**Table 6:** The changes of OCT findings in the period from 9 to 18 M follow-up angiography

	Shorter DAPT group	Longer DAPT group	*P* value
Δ Neointimal volume index, mm^3^/mm			
All potions	0.23 ± 0.29	0.19 ± 0.27	0.56
Proximal	0.26 ± 0.33	0.18 ± 0.31	0.08
Mid	0.13 ± 0.37	0.15 ± 0.39	0.26
Distal	0.30 ± 0.49	0.25 ± 0.57	0.57

Δ neointimal volume index defined as (neointimal volume index at 18-month follow-up) − (neointimal volume index at 9-month follow-up). Values are expressed as mean standard deviation (SD) or *n* (%)

*OCT* optical coherence tomography, *M* months

## Discussion

The main finding in this study was that interrupting DAPT after the 9-month follow-up did not influence stent strut coverage, the existence of thrombus or neointimal volume at 18-month follow-up as assessed by OCT.

Insufficient neointimal proliferation and incomplete healing were often observed in DES compared with BMS, and have been associated with late stent thrombosis in previous autopsy reports [18–20]. DAPT has thus been continued for a longer duration after DES implantation to prevent thrombotic events. A previous DAPT study supported the effect of longer DAPT administration for > 1 year in preventing these adverse events of DES [4]. On the other hand, hemorrhage is one disadvantage of continuing DAPT for longer periods.

Recently, DES have seen improvements in stent design, polymers, drug dose, and so on, creating the so-called second generation of DES. Some OCT studies have shown better stent strut coverage and vascular healing response for implanted second-generation DES than for first-generation DES at mid-term follow-up [21, 22]. This improvement allows DAPT duration to be shorter without increasing thrombotic events. In fact, first-generation DES have mainly been used in DAPT studies and most implanted DES in recent papers that have recommended shorter DAPT duration have been second-generation DES [6, 23–25]. In our study, vascular healing and appearance of thrombus after second-generation DES at mid-term follow-up were almost uniform regardless of the duration of DAPT. In the shorter DAPT group, neointimal volume increase due to thrombus formation in the period from 9-month to 18-month follow-up were not observed in these serial OCT examinations. Our data support shorter DAPT duration as acceptable, especially for second-generation DES.

As for changes in OCT findings during the period from 9- to 18-month follow-up, percentage stent strut coverage and neointimal volume increased in both groups numerically, but not statistically significant. The rate of covered struts in our study was not markedly different from that in previous papers (87.5–99.7%) on mid-term OCT after second-generation DES implantation [21, 26, 27]. These results showed that neointimal proliferation continued overtime in the mid-term after second-generation DES implantation. However, the increase in neointimal volume was very low and was considered to be of little clinical relevance. These findings suggest favorable neointimal proliferation for second-generation DES.

This study had several limitations. First, it was a single-center, retrospective, observational study with no randomization. Second, the sample size was relatively limited, raising the possibility of selection bias. These limitations meant that no definitive conclusions have been reached. Third, we did not evaluate patients with target lesion failure or adverse events during follow-up. Finally, the follow-up period was limited to 18 months. We therefore could not assess correlations between OCT findings and clinical outcomes such as stent thrombosis or late catch-up ISR. Long-term follow-up in a larger population is needed to confirm our results.

## Conclusion

Interrupting DAPT at 9 months after second-generation DES implantation might not affect the development of in-stent thrombus, neointimal proliferation or stent strut coverage compared with DAPT continued for up to 18 months.
